# Recommendations for long-term outcomes in sepsis and septic shock: a comparison between Japanese and international guidelines

**DOI:** 10.1186/s40560-022-00599-3

**Published:** 2022-02-05

**Authors:** Yusuke Kawai, Osamu Nishida

**Affiliations:** 1 Department of Nursing, Fujita Health University Hospital, 1-98 Dengakugakubo, Kutsukake-cho, Toyoake, Aichi 470-1192 Japan; 2 Department of Anesthesiology and Critical Care Medicine, Fujita Health University School of Medicine, 1-98 Dengakugakubo, Kutsukake-cho, Toyoake, Aichi 470-1192 Japan

**Keywords:** The International Guidelines for Management of Sepsis and Septic Shock 2021, The Japanese Clinical Practice Guidelines for Management of Sepsis and Septic Shock 2020, Long-term outcomes, Patient- and family-centered care, Health care systems

## Abstract

The International Guidelines for Management of Sepsis and Septic Shock 2021 and the Japanese Clinical Practice Guidelines for Management of Sepsis and Septic Shock 2020 share a common issue on long-term outcomes of patients with sepsis and septic shock and their families; however, the focus of the clinical questions and recommendations between the two guidelines varies. Although this may be due to differences in medical resources and healthcare systems between countries and regions, the essence of providing continuous patient- and family-centered care remains unchanged, and both guidelines can be utilized to provide the best practices to improve long-term outcomes.

## Dear Editor,

We would like to address some differences between the recently published the International Guidelines for Management of Sepsis and Septic Shock 2021 (SSCG2021) [1, 2] and the Japanese Clinical Practice Guidelines for Management of Sepsis and Septic Shock 2020 (J-SSCG2020) [3] in terms of their recommendations for long-term outcomes of patients with sepsis and septic shock and their families. Although both guidelines share the same issue on long-term care and outcomes, it is interesting to note that the focus of their clinical questions (CQs) and recommendations vary; that is, SSCG2021 focuses primarily on care after intensive care unit (ICU) discharge and considers country differences such as economic aspects in determining its CQs and recommendations, whereas J-SSCG2020 focuses primarily on care in the ICU in Japan.

First, regarding shared decision-making, J-SSCG2020 focuses on end of life. This may be due to the fact that while the United States, United Kingdom, Australia, Taiwan, South Korea, and other countries have legislated advance directives and are developing advance care planning (ACP), Japan has no laws related to advance directives and has little awareness concerning ACP. As of December 2017, only 3.3% of the general public and approximately 20% of physicians and nurses were familiar with ACP [4]. Although SSCG2021 also recommends shared decision-making, its focus is not on end of life but mainly on post-ICU and hospital discharge planning. This recommendation of SSCG2021 is potentially useful in Japan because approximately 80% of the Japanese citizens have expressed their desire in making their own decisions regarding their care plan after consultation or explanation with the physicians [5].

Second, in addition to shared decision-making, SSCG2021 describes many CQs and recommendations for transition of care and post-ICU care. However, J-SSCG2020 only provides information in a background question. This may be because Japan has a larger number of hospital beds per 1000 population than most other countries [6] and because the consolidation of medical care is not as advanced as in some other countries due to the scattering of medical resources. In Japan, the average length of hospital stay is more than twice that of other countries [6] and primary care has not yet been institutionalized [7]. According to a 2021 survey of ICUs in Japan, the implementation rates of ICU medical professionals following post-ICU patients on the wards, outpatient ICU follow-up clinics, and sharing of post intensive care syndrome (PICS) information between hospitals and medical institutions (e.g., other hospitals, clinics, and nursing homes) post-discharge were low, at 32.7%, 3.6%, and 19.1%, respectively [8]. However, as Japan's medical policy will rapidly promote the functional division/strengthening and cooperation of medical institutions in the future [9], these recommendations in SSCG2021 will become even more important in Japan to continuously provide consistent medical care to patients.

Third, SSCG2021 describes a CQ and recommendation on economic and social support, whereas J-SSCG2020 does not. This may be due to the different methodologies used to develop the two guidelines. In the long-term outcomes and goals of care group of SSCG2021, the questions were developed by a multi-country panel that included at least one representative from a low- or middle-income country, and 11 patient and family representatives from different countries and backgrounds helped to develop and rate the outcomes for each question, reviewed evidence summaries, and provided input on recommendations. Therefore, SSCG2021 discussed the adaptation of the recommendations to not only high-income settings but also to low- and middle-income settings. On the other hand, the ICU-acquired weakness and early rehabilitation group and the patient- and family-centered care group of J-SSCG2020 were similar in that they included patient representatives, but differed in that they were composed of members only from Japan. The health care system in Japan guarantees free access to medical care with low out-of-pocket payments to all Japanese citizens through the universal health insurance system that is subsidized by public funds [7, 10].

The two guidelines have many CQs and recommendations for long-term outcomes. SSCG2021 describes a total of 20 recommendations from 12 CQs, and J-SSCG2020 describes a total of seven recommendations from nine CQs, including two background questions. However, the recommendations in SSCG2021 include eight best practice statements, and in all recommendations using GRADE methodology in both guidelines, the strength of the recommendation is weak, and the quality of evidence is low or very low. Recommendations for long-term outcomes of these guidelines remain difficult to determine as their evaluation involves a wide range of management protocols and assessment of various interventions for complex patients in culturally and socioeconomically diverse settings. Although there are few recommendations that overlap in J-SSCG2020 and SSCG2021, the essence of providing continuous patient- and family-centered care to improve long-term outcomes and quality of life of patients and their families remains unchanged. It is recommended that both guidelines be utilized to provide the best practices.

## Data Availability

Not applicable.

## Abbreviations

ACP: Advance care planning
CQ: Clinical question
ICU: Intensive care unit
J-SSCG2020: The Japanese Clinical Practice Guidelines for Management of Sepsis and Septic Shock 2020
PICS: Post intensive care syndrome
SSCG2021: The International Guidelines for Management of Sepsis and Septic Shock 2021
